# Antimicrobial Characteristics of Lactic Acid Bacteria Isolated from Homemade Fermented Foods

**DOI:** 10.1155/2018/5416725

**Published:** 2018-12-30

**Authors:** Dayong Ren, Jianwei Zhu, Shengjie Gong, Hongyan Liu, Hansong Yu

**Affiliations:** ^1^College of Food Science and Engineering, Jilin Agricultural University, Changchun 130118, China; ^2^College of Chinese Herbal Medicine, Jilin Agricultural University, Changchun 130118, China

## Abstract

*Objective*. Lactic acid bacteria (LAB) were isolated from fermented foods, such as glutinous rice dough, corn noodle, chili sauce, potherb mustard pickles, and stinky tofu, in northeast China. LAB strains with antimicrobial activities were screened, and seven of these* Lactobacillus *strains were identified as* L. plantarum*,* L. pentosus*, and* L. paracasei* through 16S rRNA gene analysis. After the supernatant of LAB was treated with proteinase K, pepsin, and papain, their antibacterial effect almost disappeared. Most strains with antibacterial activities were highly resistant to heat (65°C–121°C), acidity (pH 2–6), and alcohol. The antimicrobial effect of most strains treated with the Tween-80 surfactant was significantly reduced, and the antibacterial property of T4 was even lost. Ammonium sulfate precipitation, PCR, and nanoLC-ESI-MS/MS results confirmed that T8 produced antibacterial substances belonging to a protein family, and its zone of inhibition against pathogens significantly increased (>13 mm). In bacterial growth inhibition experiments, the colony count of* Staphylococcus aureus* was up to 10^15^ CFU/mL in the 3⁎de Man, Rogosa, and Sharpe (MRS) group, and this value was more than that in the 3⁎S6 supernatant group (10^12^ CFU/mL) and the control group (10^10^ CFU/mL) at 12 h. This study provided a basis for the selection of antimicrobial peptides and the development and utilization of LAB.

## 1. Introduction

Food-borne diseases associated with the consumption of fresh and minimally processed agricultural products have resulted in remarkable outbreaks and caused health problems. The side effects of the improper use of artificial preservatives and antibiotics have become serious. In the food industry, traditional sterilization methods involve the use of chemical sanitizers or high-temperature heating sterilization. However, in this way, harmful bacteria are incompletely eradicated, and organoleptic qualities decline [1]. As such, current studies aim to extend shelf life and antibacterial property by using antibacterial substance from microorganisms via [2].

*Lactobacillus* is widely used as probiotics in fermented foods. In vitro analysis found that lactic acid bacteria (LAB) have antioxidant effects and can chelate ferrous ions and degrade nitrite and cholesterol [3, 4]. LAB are natural microbes, and their metabolites are generally regarded as safe [5]. For example, nisin, which is an antimicrobial preservative, is the only allowed food preservative from the* lactococcus lactis *to prevent the growth of specific pathogens and spoilage caused by organisms and bacteria. LAB metabolic products, such as acid, hydrogen peroxide, and bacteriocin, can inhibit some bacteria and fungi [6]. Some strains do not elicit antimicrobial effects because their metabolic production is insufficient or minimal [7]. Therefore, LAB with high antibacterial activities should be screened, and their antibacterial components should be analyzed.

LAB isolated from yogurt have been screened and functionally analyzed, and studies have revealed that their antibacterial effects are not evident. However, LAB isolated from homemade fermented foods should be examined. In this experiment, five kinds of homemade fermented foods in Northeast China were selected as raw materials. Considering that LAB have these effects in vitro and that their metabolites may target and play a role in the competitive exclusion of pathogens, we should screen bacteria that produce numerous antimicrobial peptides and acids that are good biopreservatives for pickled products [8]. Therefore, our study aimed to extend the screening of LAB and to obtain novel and good strains.

## 2. Materials and Methods

### 2.1. Bacterial Isolation and Identification

*Salmonella enterica* (ATCC14028),* Staphylococcus aureus* (ATCC 6538p),* Escherichia coli* (ATCC 8739), and* Bacillus cereus *Frankland (CICC 20551) used in the experiment were purchased from the China Center of Industrial Culture Collection (CICC).

A total of 231 strains were randomly isolated from fermented foods, namely, nianmianzi (glutinous rice dough), tangzimian (corn noodle), chili sauce, potherb mustard pickles, and stinky tofu, through serial dilution in MRS agar (Qingdao Hopebio Co.) and LAB purification twice [9]. H, T, L, and S were the abbreviations used for the fermented foods and the number of isolates corresponding to the strain name. Plate count agar was utilized to monitor viable bacteria for the reserve concentration and stored at −80°C in MRS with glycerol before use. LAB were identified using a 16s rRNA gene with the primers 27F (5′-AGAGTTTGATCCTGGCTCAG-3′) and 1492R (5′-TACGGYTACCTTGTTACGACTT-3′) [10]. Thereafter, sequencing was performed in Basic Local Alignment Search Tool in the EzTaxon-e database with a sequence-matching program.

### 2.2. Culture Conditions

All of the isolated strains were incubated in MRS broth (37°C, 48 h). The second culture was incubated (1%, v/v) for 1 day, and the viable cell count was adjusted to 10^8^ colony forming units (CFU)/mL. The strains were stored at −20°C in all of the experiments.

*Salmonella*,* S. aureus*,* E. coli*, and* Bacillus cereus Frankland* were grown for 24 h and incubated in 1% inoculation at 37°C with shaking at 200 rpm in CM0002 broth (CICC). The second culture was incubated overnight at 37°C and 200 rpm.

### 2.3. Screening for the Antimicrobial Activity

The antimicrobial activity of the LAB supernatant was analyzed. All of the supernatants were precipitated via centrifugation at 4000 ×* g* for 20 min at 4°C when 1% inoculum was incubated for 24, 48, and 72 h at 37°C. pH was adjusted to 6.0 to rule out acid inhibition. All of the treated supernatants were stored at 4°C. Testing was subsequently performed against all of the indicator strains by using an Oxford cup (internal diameter of 6.0 mm) diffusion method. The spread plate method was prepared by adding the indication inocula of 100 *μ*L of 1.2 OD_600_ into the CM0002 agar plate[11, 12]. Ampicillin (25–100 *μ*g/mL, Beijing Solarbio Science & Technology) and nisin (500 *μ*g/mL, dissolved in 0.05% acetic acid/0.1 M EDTA, Shanghai Seebio Biotech, Inc.) were used as a positive control in the plate. Furthermore, 100 *μ*L of the treated supernatant was applied to an Oxford cup in the plate at 37°C for 24 h.

### 2.4. Effect of Enzymes on the Antimicrobial Activity

Enzymes were added to the supernatant of the selected strains to evaluate their effect on bacteriocin-like inhibitory substances. The supernatants were treated with catalase (5220 U/mg, Beijing Solarbio Science & Technology Co.) in a water bath at 25°C for 1 h, filtered, and stored at 4°C for the succeeding experiments. CaCl_2_ buffer (0.05 mol/L Tris, 5 mmol/L CaCl_2_, and pH 7.0) was added at 1 mg/mL enzymes, such as proteinase K (>30 U/mg, Beijing Solarbio Science & Technology Co.), *α*-amylase (100,000 KSB, Aobox Biotechnology), lysozyme (20,000 U/mg, Aobox Biotechnology), and papain (400 U/mg, Beijing Dingguo Changsheng Biotechnology Co.). For another experiment, citrate buffer solution was prepared (pH 3), and 1 mg/mL pepsin (250 U/mg, Beijing Solarbio Science & Technology Co.) was dispensed. All of the solutions were filter sterilized. The treated supernatants were set at 37°C for 3 h, and the mixture was boiled for 3 min to inactivate the enzymes. Bacteriostatic effects were analyzed using the Oxford cup diffusion method.

### 2.5. Antimicrobial Activity after Treatment under Varying Conditions

The treated supernatants were selected and incubated for 48 h. The influence of temperature on the antimicrobial activity of the supernatants heated at 65°C, 85°C, 100°C, and 121°C was estimated [13]. The effect of pH was determined by adjusting the pH of the supernatant to 2, 3, 4, 5, 6, 8, and 14. The supernatant was exposed to 30°C for 1 h. Finally, pH was modified to 6.0.* n*-Butanol, methanol, and ethanol were added to the supernatant at 1:9 (vol/vol) and placed at 30°C for 30 min (organic solvent from Beijing Chemical Works). Afterward, 1% (w/t) sodium citrate, potassium chloride, and Tween-80 were added to the supernatant and mixed. All of the treated solutions of the antimicrobial experiments were incubated for 24 h at 37°C by using the Oxford cup diffusion method. The residual activity of the strains was determined by observing their zone of inhibition. Sterile water and untreated samples were used as the control.

### 2.6. Ammonium Sulfate Precipitation of the Concentrated Antibacterial Components

(NH_4_)_2_SO_4_ was added to 100 mL of the supernatant of the cultured strain reaching 80% concentration (4°C, 24 h) for further purification to evaluate whether the antimicrobial components of LAB belong to a protein family[14]. The treated supernatants were then centrifuged similar to those in the preceding experiments. The supernatants were subsequently dialyzed, and the antimicrobial activities of the concentrates were determined using the Oxford cup method against* S. aureus *and* Salmonella.*

### 2.7. Inhibition of Bacterial Growth

The inhibitory effects of the supernatants were identified by adding them to the indication broth of* S. aureus* and* Salmonella*. Afterward, 1 and 3 mL of the supernatants at pH 6.0 (48 h) were added at approximately 10^7^ CFU/mL at the initial stage to 100 mL of CM0002 broth to determine the growth curves of indicators at 37°C and 200 rpm. At an interval of 2 h, the bacterial suspensions were measured at an optical density of 600 nm (OD_600_) until they were incubated for 12 h. The initial MRS, nisin–MRS (500 *μ*g/mL), ampicillin–MRS (100 *μ*g/mL), and kanamycin–MRS (100 *μ*g/mL) were prepared as described above at 37°C for 48 h as the control. Flat colony counting method was applied to determine the total number of colonies at 37°C (12–24 h).

### 2.8. Identification of Antimicrobial Peptides

The target gene of seven strains was amplified using primers (forward 5′-ATGAAAAAATTTCTAGTTTTGCGTGAC-3′andreverse 5′-CTATCCGTGGATGAATCCTCGGACAGC-3′) via PCR. PCR was performed in accordance with the ExTaq (Takara) reaction protocol as follows: 95°C for 2 min, followed by 35 cycles at 95°C for 30 s, annealing at 55°C for 45 s, and extension at 72°C for 30 s. The T8 sample was further analyzed using nanoLC-ESI-MS/MS (ProtTech, Suzhou, China).

### 2.9. Data Analysis

All of the experiments involved three randomly selected replicates per treatment. SPSS was used to analyze the data through one-way ANOVA with a significance level of 0.5%.

## 3. Results and Discussion

### 3.1. Antimicrobial Activity Evaluation

The preliminary effect of the antimicrobial activity of the strains was determined via the diffusion method with 1 M HCl/NaOH to remove the acid that could inhibit the production of pathogenic bacteria in the supernatant. Thirty-five strains presented antimicrobial activity against* S. aureus *(Table 1). Eleven strains also elicited a strong antibacterial effect with an inhibition zone of more than 8.9 m. The inhibition zones of S6, L2, T8, and H9 were greater than 9.2 mm. Similar conditions were observed in the* Lactobacillus* inhibition of* Salmonella*. Eight strains covered an inhibition zone of greater than 9 mm. In comparison with the control group and the treatment group against* Bacillus cereus*, pathogenic bacteria slightly grew in the middle of the inhibition zone in the treatment group. Some spores might grow in the pathogenic* B. cereus *congenicstrain, and LAB were not well inhibited. As such,* B. cereus* was excluded from further investigation. Most strains did not inhibit* E. coli *except L2, L11, L19, T4, T8, T30, H9, H12, and S8 strains with an inhibition zone of 8 mm. Although most strains were limited against one pathogen in our study, seven strains displayed a broad-spectrum bacteriostatic effect. In general, the inhibitory effect of the isolated antibacterial strains on* S. aureus *and* Salmonella *was greater than that on* E. coli *and* Bacillus*. The results suggested that the concentration and type of the produced antibacterial substance differed from those of LAB. Thus, different LAB showed various degrees of inhibitory activity against pathogenic bacteria. Lanhua Yi [15] revealed that* Lactobacillus *can be enhanced to inhibit pathogenic bacteria selectively and confirmed that* L. coryniformis *XN8 exhibits a broad-spectrum antimicrobial activity and induces a strong antibacterial effect against* S. aureus*. This finding confirmed the characteristics of the selective inhibition of LAB [16].

pH was roughly used to determine the acidity of the produced* Lactobacillus.* Table 1 shows that the amount of acid (pH < 3.7) secreted by two strains, namely, T30 and S6, was higher than that produced by other strains. This finding indicated the varying transport regulation and metabolism of the lactose system of LAB and the acid production ability of the different strains. Therefore, S6 shows potential as a biopreservative and fermentation agent in fermented food production[17].

Seven strains, namely, L2, L16, L19, T4, T8, H9, and S6, and the indicator bacteria, namely,* S. aureus* and* Salmonella*, were examined for further relevant experimentation.

### 3.2. Strain Identification

The seven isolates with antimicrobial activities were identified using a 16s rRNA gene. The results were compared with the data in the EzTaxon-e database.  Table 2 shows three* Lactobacillus* species, namely,* L. plantarum *(L2, L16, T4, and T8),* L. pentosus *(L19 and S6), and* L. paracasei *(H9). The strains reached more than 99% similarity.

### 3.3. Identification of Antimicrobial Components

Most strains lost their antimicrobial activity after acid was removed. L19 slightly decreased the antibacterial effect of the inhibited bacteria after catalase treatment was administered. This finding suggested that the main antimicrobial effect of some strains is dependent on acid and confirmed that hydrogen peroxide elicits a bacteriostatic effect [18].  Table 3 shows the bacteriostatic effect of the seven strains after enzyme treatment was administered. These supernatants of the strains were partially inactivated by using *α*-amylase and lysozyme and consequently induced to decrease their antimicrobial activities. The antimicrobial activities of the strains treated with proteinase K, pepsin, and papain were completely inactivated, but the antimicrobial activity of S6 was slightly retained after papain was administered. NatarajanDevi [19] demonstrated that bacteriocin produced by* L. sakei* GM3 isolated from goat milk is unstable after pepsin, trypsin, papain, and proteinase Kare administered. This finding suggested that the primary antimicrobial activity of the seven strains was also dependent on peptides after acid and catalase were removed. Moreover, the results showed that antimicrobial substances may contain a carbohydrate that promotes inhibition to a certain extent.

### 3.4. Effects of Temperature, pH, Additives, and Organic Compounds on Antimicrobial Components

After the treatments were administered at different temperatures, their effects on the antimicrobial activity of LAB were stable at a low temperature for 30 min (Table 4). Similarly, bacteriocins produced by the strains isolated from salpico are thermostable at 100°C for 20 min[20]. The bacteriocins produced by* L. bulgaricus* BB18 and* L. lactis* BCM5 were highly stable at high temperatures, and their antimicrobial activities were retained after 60 min at 100°C. However, the antimicrobial activity of L16 and pathogenic growth in the zone against the indicator bacteria was reduced to some extent at 121°C for 20 min. Most antimicrobial components remained stable at high temperatures. The molecular weight of antimicrobial peptides, which are secondary protein structures (*α*-helix, *β*-folding, *β*-rotation angle, and random crimp), is between 3 and 10 kDa. Their low molecular weight and secondary structure may lead to the high-temperature resistance of most antimicrobial peptides [21]. This finding indicated that the antibacterial components of LAB could be applied as biological preservatives for high-temperature treatments of food.

Most strains were inhibited on two indications when three types of organic solvents were added to the supernatants (Table 4), and this observation was consistent with that reported by Natarajan Devi [19], who demonstrated that bacitracin can be soluble in organic solvents. However, the antibacterial effect was reduced to some extent when methanol was added to T4 and L19 supernatants possibly because the surface structure of various antimicrobial agents caused intolerance to methanol.

The antimicrobial activity of the supernatant against the indicator was stable within a wide pH range (2.0–6.0). The antimicrobial activity of LAB was also retained at pH 8 for 30 min, but the inhibitory effect of S6 was evidently reduced. The antimicrobial activities at pH 14 were completely lost, and this finding was consistent with* Pediococcus pentosaceus* bacteriocin ALP57 that loses its antimicrobial activity at pH 12[22]. In our study, high antimicrobial activities were detected at low pH.

All of the supernatants were treated with additives, such as sodium citrate, potassium, and surfactants, to verify the effect of food additives and other chemicals on the antibacterial components of LAB (Table 4). LAB showed a stable antimicrobial activity against the indicator bacteria treated with sodium citrate and potassium. After Tween-80 was added, the antimicrobial activity remarkably differed, and T4 lost its antimicrobial activity. Priscilia Y [23] reported that the antimicrobial activity of* Lactobacillus* spp. isolated from Mexican Cocido cheese against* S. aureus*,* Listeria innocua*,* E. coli*, and* S. typhimurium* decreases when anionic compounds are added. However, the bacteriostatic effect of bacteriocin CM3 is stable when different surfactants are added. These behaviors could be explained by LAB from different sources producing bacteriocin-like substances (BLS), whose surface structure varies and consequently results in different sensitivities to Tween-80. Overall, the different surface structures of the BLS affect their antimicrobial activity, and their substance tolerance varies.

L2, S6, and T8 strains were selected for further experiments based on our results.

### 3.5. Analysis of the Antibacterial Components after Purification

**Figure 1:**
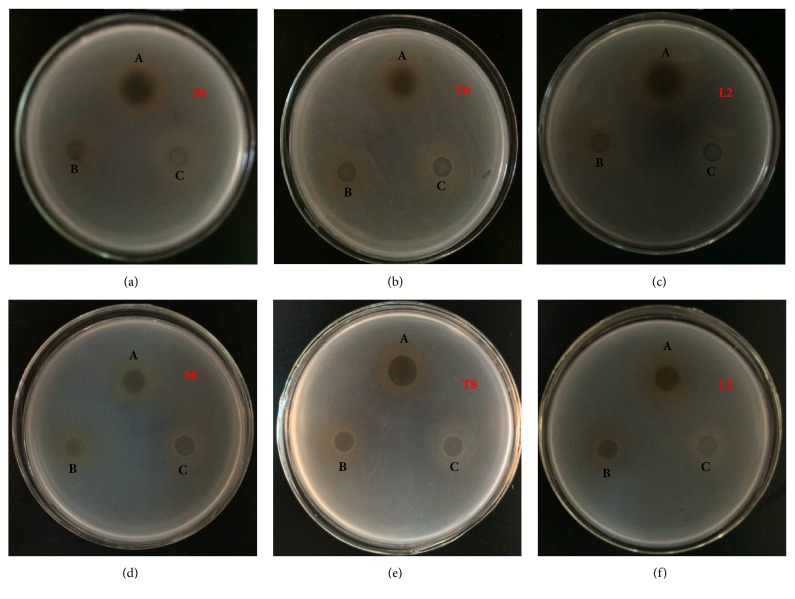
Crude extraction test after preliminary purification against* S. aureus *(a, b, and c) and* Salmonella *(d, e, and f). A: sample concentration. B: MRS concentration. C: water in each figure.

In the experiment, the concentrate that inhibited the indicator was enhanced after ammonium sulfate precipitated, revealing that the antimicrobial components of these strains were proteins (Figure 1). The comparisons were drawn between the inhibition zones of the untreated and concentrated samples. All of the inhibition zones of the concentrate against* S. aureus* were enlarged by >12 mm (Figures 1(a), 1(b), and 1(c)). The inhibition zone of S6 reached 15.22±0.13 mm. The concentrations of T8 and L2 remarkably inhibited* Salmonella *(Figure 1), and the inhibition zone of T8 was more than 13 mm. Overall, the concentrate could enhance the antimicrobial activity, and the observation was the same as that in previous studies, which showed that the ability of pure plantaricin NC8 to inhibit food-borne pathogens is evidently higher than that of the untreated group [24]. Ammonium sulfate precipitate produced from different strains elicited varying inhibitory effects against bacteria, suggesting that LAB selectively inhibit pathogens, and their antimicrobial components belong to a protein family [15].

S6 and T8 were selected for the subsequent experiments because the concentrated T8 significantly enhanced the bacteriostatic effect of the two pathogenic bacteria, and S6 inhibited* S. aureus *to a greater extent than other strains.

### 3.6. Effect of Various Factors on the Growth Curve of the Indicator Bacteria

**Figure 2:**
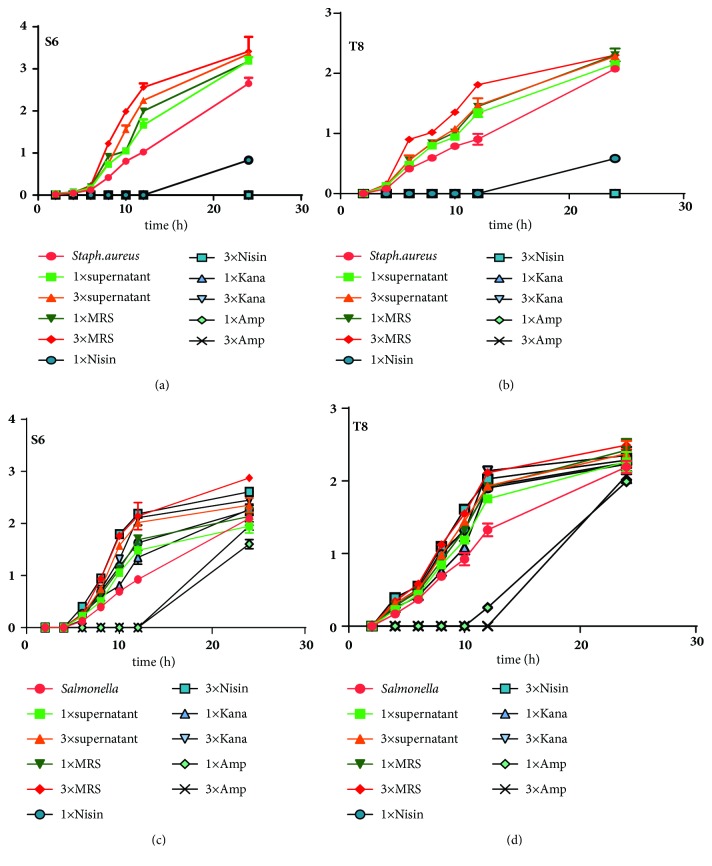
Effects of various factors on the growth of strains.* S. aureus *(a, b) and* Salmonella *(c, d) are the indicator bacteria.

Figure 2 shows the influence of various factors on the growth of pathogenic bacteria. Lanhua Yi [15] reported that LAB can inhibit bacterial growth to some extent. The antibiotic–MRS completely inhibited the non-drug-resistance genes of* S. aureus*. However, OD_600_ and the population of the indicator in the MRS group were higher than those of the experimental group with the same volume until 12 h. Meanwhile, the population of* S. aureus* in 3*∗*supernatant (S6) ranged from 12 log^10^ CFU/mL to 15 log^10^ CFU/mL compared with that in 3*∗*MRS, which was significantly higher than that in the 1*∗* group as revealed by the results of the colony counting method at 12 h. The positive control group of antibiotic–MRS also favors the growth of* Salmonella* to some extent [25, 26]. This finding is consistent with that described in a recent work, which showed the resistance of* Salmonella* to gentamicin, ciprofloxacin, aminoglycosides, and tetracycline [27, 28] (Figures 2(c) and 2(d)). This phenomenon remarkably altered the OD_600_ and the colony number of* Salmonella* in the antibiotic–MRS group. In this study, the OD and clump count revealed that the supernatant or the antibiotic could decrease the growth of the indicator bacteria compared with that of the control group with MRS.

### 3.7. Identification of Bacteriocin

**Figure 3:**
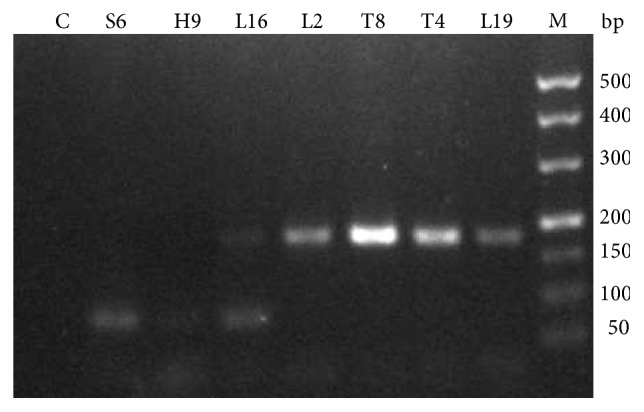
Agarose gel electrophoresis of the PCR product. Line L19–S6: PCR result of the seven strains. Line C: negative control.

PCR showed a 150–200 bp DNA fragment in Figure 3 (L19, L16, L2, T4, and T8). Sequence analysis revealed that the target DNA fragment reached 100% similarity compared with that of PlnF in the National Center for Biotechnology Information. nanoLC-ESI-MS/MS indicated that the peptide had a molecular weight of 5729.03 Da (Table 5). In comparison with UniProt, the submitted peptide was bacteriocin peptide plnF, whose relative abundance reached 97.8%. Albert [29] reported the presence of plantaricin-encoding genes, such as plnA, plnB, plnE/F, and plnF, in LAB.

## 4. Conclusions

In vitro studies showed that the antibacterial components of seven LAB isolated from nianmianzi, tangzimian, chili sauce, potherb mustard pickles, and stinky tofu were resistant to certain temperature ranges (65°C–121°C), acidity (pH 2–6), alcohol, and some additives. After preliminary enrichment was performed using ammonium sulfate, the bacteriostatic effect of* L. pentosus* S6 against* S. aureus* was up to 15.22±0.13 mm, the bacteriostatic effect of* L. plantarum* T8 increased to 13.08±0.15 mm, and the inhibition zone of T8 against* Salmonella* increased to 13.37±0.19 mm. These findings demonstrated that the crude extract of T8 elicited good broad-spectrum antibacterial effects. The results of the growth inhibition experiment of pathogenic bacteria confirmed that LAB had certain supernatant fluid that inhibited bacterial growth. nanoLC-ESI-MS/MS and PCR results indicated that the bacteriocin peptide plnF existed in T8. Our results provided a basis for performing future studies on the heterogeneous expression of antimicrobial peptides and screening other suitable culture media for LAB growth of LAB.

## Figures and Tables

**Table 1:** Antimicrobial activity of the supernatant of the strains against pathogenic bacteria.

Strain	pH (48 h)	Inhibition zone (mm) of S. aureus			Inhibition zone (mm) of Salmonella		
		24 h	48 h	72 h	24 h	48 h	72 h
T4	3.88±0.01	9.13±0.12	9.17±0.15	9.07±0.06	9.09±0.07	9.04±0.04	9.03±0.10
T5	3.82±0.02	8.03±0.15	8.13±0.06	8.17±0.12	8.67±0.08	8.71±0.08	8.69±0.07
T6	3.76±0.01	8.00±0.20	8.03±0.06	8.33±0.35	8.18±0.12	8.14±0.04	8.19±0.11
T8	3.72±0.04	9.27±0.25	9.23±0.05	9.20±0.20	9.03±0.13	9.05±0.13	9.03±0.06
T12	3.76±0.03	-	8.46±0.12	8.26±0.21	7.85±0.18	8.16±0.10	8.09±0.04
T13	4.06±0.04	-	7.83±0.21	7.97±0.12	9.07±0.11	8.98±0.12	9.09±0.09
T18	3.88±0.01	8.89±0.15	8.87±0.08	8.73±0.31	8.48±0.06	8.59±0.11	8.44±0.14
T19	3.96±0.06	7.70±0.26	7.93±0.12	7.90±0.10	-	-	-
T20	4.02±0.16	7.83±0.02	7.85±0.01	8.13±0.05	8.33±0.05	8.24±0.08	8.34±0.15
T24	3.81±0.04	9.00±0.17	8.87±0.13	8.93±0.06	-	-	-
T25	3.95±0.02	-	7.86±0.02	7.94±0.05	8.70±0.11	8.64±0.05	8.61±0.03
T26	3.92±0.08	8.66±0.03	8.74±0.05	8.61±0.03	-	-	-
T27	3.97±0.01	7.50±0.26	7.37±0.23	7.40±0.27	7.92±0.09	8.01±0.15	7.87±0.03
T28	3.87±0.03	8.06±0.11	8.70±0.10	8.80±0.26	8.66±0.17	8.72±0.07	8.68±0.18
T30	3.69±0.03	8.63±0.06	8.70±0.17	8.64±0.07	8.51±0.16	8.45±0.08	8.52±0.19
T52	3.87±0.02	8.13±0.07	8.17±0.08	8.08±0.08	-	-	-
L1	3.90±0.01	8.20±0.02	8.16±0.03	8.12±0.08	7.94±0.21	7.85±0.09	7.83±0.05
L2	3.86±0.04	9.33±0.08	9.28±0.03	9.25±0.15	9.29±0.06	9.40±0.26	9.23±0.10
L8	3.88±0.02	9.22±0.15	9.28±0.12	9.21±0.16	8.38±0.14	8.39±0.13	8.31±0.14
L11	3.82±0.01	-	7.79±0.02	7.83±0.01	-	-	-
L12	3.95±0.03	-	-	-	7.89±0.02	7.96±0.04	7.91±0.08
L13	3.97±0.06	8.23±0.03	8.22±0.03	8.17±0.07	8.88±0.08	8.85±0.06	8.83±0.08
L14	3.88±0.02	8.29±0.06	8.18±0.02	8.15±0.04	8.35±0.18	8.42±0.13	8.36±0.04
L15	3.90±0.05	8.42±0.03	8.41±0.08	8.37±0.08	7.03±0.26	7.72±0.37	7.65±0.19
L16	3.84±0.02	9.18±0.09	9.17±0.05	9.17±0.10	9.06±0.07	9.16±0.14	9.22±0.25
L18	3.99±0.03	-	-	-	8.29±0.05	8.30±0.05	8.21±0.13
L19	3.79±0.01	9.23±0.06	9.26±0.06	9.14±0.04	9.17±0.08	9.19±0.02	9.11±0.06
L20	3.99±0.04	-	8.27±0.08	8.21±0.04	8.23±0.07	8.34±0.06	8.31±0.02
L21	3.85±0.05	8.31±0.15	8.27±0.05	8.39±0.03	7.89±0.14	7.81±0.05	7.82±0.09
L25	3.91±0.02	8.65±0.05	8.57±0.11	8.54±0.02	8.22±0.04	8.16±0.08	8.13±0.04
L27	3.87±0.07	-	7.32±0.09	7.32±0.03	8.23±0.07	8.77±0.07	8.50±0.00
L31	4.12±0.10	8.15±0.09	8.09±0.04	8.15±0.06	7.75±0.39	8.29±0.08	8.43±0.09
S6	3.67±0.04	9.42±0.18	9.39±0.09	9.42±0.10	9.03±0.06	9.07±0.03	9.06±0.02
S8	3.90±0.11	9.12±0.03	9.20±0.13	9.09±0.09	8.18±0.09	8.27±0.10	8.23±0.03
S13	3.92±0.05	8.07±0.06	8.05±0.05	8.08±0.11	-	-	-
H9	3.92±0.04	9.38±0.09	9.32±0.09	9.27±0.08	9.08±0.07	9.17±0.09	9.03±0.06
H12	3.88±0.03	8.50±0.05	8.48±0.03	8.56±0.07	8.23±0.03	8.23±0.05	8.26±0.17
H27	3.97±0.02	8.06±0.04	8.10±0.08	8.04±0.01	7.90±0.03	8.08±0.10	7,97±0.05
nisin		14.53±0.15	14.43±0.28	14.48±0.07	12.73±0.16	12.65±0.05	12.73±0.14
ampicillin		19.21±0.07	19.18±0.13	19.20±0.10	14.96±0.11	15.05±0.10	15.05±0.05

**Table 2:** Identification of strains.

Strain	Isolation source	Identified	%Similarity
L2	chili sauce	*Lactobacillus plantarum*	100%
L16	chili sauce	*Lactobacillus plantarum*	100%
L19	chili sauce	*Lactobacillus pentosus*	99.79%
T4	corn noodle	*Lactobacillus plantarum*	100%
T8	corn noodle	*Lactobacillus plantarum*	100%
H9	glutinous rice dough	*Lactobacillus paracasei*	100%
S6	stinky tofu	*Lactobacillus pentosus*	100%

**Table 3:** Effects of antimicrobial after treatment with enzymes.

Enzyme	Strains						
	L2	L16	L19	T4	T8	H9	S6
Proteinase K	-	-	-	-	-	-	-
Pepsin	-	-	-	-	-	-	-
Papain	-	-	-	-	-	-	+
*α*-Amylase	+++	++	+	++	++	+++	+++
Lysozyme	++	++	++	+	++	++	++

ps: “-”:inhibition zone < 6 mm; “+”:inhibition zone: 6–8 mm; “++”:inhibition zone: 8–9 mm; “+++”:inhibition zone > 9 mm.

**Table 4:** Residual antimicrobial activity of *Lactobacillus* treated under different conditions.

Treatment concentration	Strains						
	L2	L16	L19	T4	T8	H9	S6
Temperature/time							
65°C/30 min	+++	+++	+++	+++	+++	+++	+++
85°C/30 min	+++	+++	+++	+++	+++	+++	+++
100°C/30 min	+++	+++	+++	+++	+++	+++	+++
121°C/20 min	+++	+	++	++	++	+++	++
pH							
2	+++	+++	+++	+++	+++	+++	+++
3	+++	+++	+++	+++	+++	+++	+++
4	+++	+++	+++	+++	+++	+++	+++
5	+++	+++	+++	+++	+++	+++	+++
8	++	++	++	++	+++	++	+
14	-	-	-	-	-	-	-
Organic solvent							
Methanol 10% (vol/vol)	++	++	+	++	++	+++	++
Ethanol 10% (vol/vol)	+++	+++	+++	+++	+++	+++	+++
n-Butanol 10% (vol/vol)	+++	+++	+++	+++	+++	+++	+++
Additive							
Potassium chloride 1% (wt/vol)	+++	++	+++	++	++	++	+++
Sodium citrate 1% (wt/vol)	+++	++	++	++	+++	+++	+++
Tween-80 1% (wt/vol)	+	+	++	-	+	+	++

ps: “-”:inhibition zone < 6 mm; “+”:inhibition zone: 6–8 mm; “++”:inhibition zone: 8–9 mm; “+++”:inhibition zone > 9 mm.

**Table 5:** nanolc-ESI-MS/MS.

Protein Mass	No. of Peptides	Sequence Header	Link	Relative Abundance	Probability
5729.03	87	Bacteriocin peptide PlnF OS = Lactobacillus plantarum (strain ATCC BAA-793 / NCIMB 8826 / WCFS1) GN=plnF	F9UU07	97.8%	99.0%

## Data Availability

Most of the data about antimicrobial activity have been showed in Table 1; and according to the partial data, we draw Figure 2. Please feel free to contact Dayong Ren when you need the whole database.

## Abbreviations

MRS: de Man, Rogosa, and Sharpe
BLS: Bacteriocin-like substances
EDTA: Ethylene diamine tetraacetic acid
CFU/mL: Colony forming unit per milliliter
*S. aureus:*: * Staphylococcus aureus*
*E. coli:*: * Escherichia coli.*
